# Grazing livestock are exposed to terrestrial cyanobacteria

**DOI:** 10.1186/s13567-015-0143-x

**Published:** 2015-02-25

**Authors:** Bruce C McGorum, R Scott Pirie, Laura Glendinning, Gerry McLachlan, James S Metcalf, Sandra A Banack, Paul A Cox, Geoffrey A Codd

**Affiliations:** Royal (Dick) School of Veterinary Studies and Roslin Institute, University of Edinburgh, Roslin, EH25 9RG Scotland UK; The Institute for Ethnomedicine, 240 East Deloney Avenue, Jackson, WY 83001-3464 USA; Biological and Environmental Sciences, School of Natural Sciences, University of Stirling, Stirling, FK9 4LA Scotland UK

## Abstract

While toxins from aquatic cyanobacteria are a well-recognised cause of disease in birds and animals, exposure of grazing livestock to terrestrial cyanobacteria has not been described. This study identified terrestrial cyanobacteria, predominantly *Phormidium* spp., in the biofilm of plants from most livestock fields investigated. Lower numbers of other cyanobacteria, microalgae and fungi were present on many plants. Cyanobacterial 16S rDNA, predominantly from *Phormidium* spp., was detected in all samples tested, including 6 plant washings, 1 soil sample and ileal contents from 2 grazing horses. Further work was performed to test the hypothesis that ingestion of cyanotoxins contributes to the pathogenesis of some currently unexplained diseases of grazing horses, including equine grass sickness (EGS), equine motor neuron disease (EMND) and hepatopathy. *Phormidium* population density was significantly higher on EGS fields than on control fields. The cyanobacterial neurotoxic amino acid 2,4-diaminobutyric acid (DAB) was detected in plant washings from EGS fields, but worst case scenario estimations suggested the dose would be insufficient to cause disease. Neither DAB nor the cyanobacterial neurotoxins β-N-methylamino-L-alanine and N-(2-aminoethyl) glycine were detected in neural tissue from 6 EGS horses, 2 EMND horses and 7 control horses. *Phormidium* was present in low numbers on plants where horses had unexplained hepatopathy. This study did not yield evidence linking known cyanotoxins with disease in grazing horses. However, further study is warranted to identify and quantify toxins produced by cyanobacteria on livestock fields, and determine whether, under appropriate conditions, known or unknown cyanotoxins contribute to currently unexplained diseases in grazing livestock.

**Electronic supplementary material:**

The online version of this article (doi:10.1186/s13567-015-0143-x) contains supplementary material, which is available to authorized users.

## Introduction

Cyanobacteria are a diverse group of ubiquitous environmental bacteria which can produce a wide array of toxins (cyanotoxins). While exposure to cyanotoxins from aquatic cyanobacterial blooms, scums and mats is a well-recognised cause of neurologic and hepatic disease in birds and animals [1], exposure of grazing livestock to terrestrial, as opposed to aquatic, cyanobacteria has not been previously described. We hypothesised that grazing livestock are exposed to *Phormidium* spp. since this cyanobacterial genus can grow on turf grasses and golf courses, particularly in favourable weather conditions and following fertiliser application [2-6]. Cyanobacteria including *Phormidium* spp. can produce a wide range of hepato-, neuro- and dermotoxins which cause disease in animals exposed to aquatic cyanobacterial blooms [7-10]. While exposure to anatoxin-a from the benthic cyanobacterium *Phormidium favosum* has been associated with canine neurotoxicosis [9], toxins from terrestrial *Phormidium* spp. have not been previously definitively associated with disease. We hypothesised that cyanotoxins from terrestrial *Phormidium* spp. may trigger or cause currently unexplained diseases of grazing livestock such as equine grass sickness (EGS), a frequently fatal multi-system neuropathy affecting grazing horses, equine motor neuron disease (EMND) and idiopathic hepatopathy. The aims of this study were to: (a) identify and enumerate *Phormidium* filaments in washings of the biofilm on plants collected from livestock-grazing fields; (b) evaluate spatial and temporal variation in the density of *Phormidium* filaments; (c) determine whether *Phormidium* filaments can be detected by microscopy in gastrointestinal contents from grazing horses; and (d) use a genomic approach to identify cyanobacteria in plant washings, equine ileal contents and soil. Additionally, a preliminary investigation of the potential association of cyanotoxins and EGS, EMND and hepatopathy was performed by: (a) comparing the density of *Phormidium* filaments on plants from horse fields immediately after an occurrence of EGS with that of plants from control horse fields; (b) quantifying the cyanobacterial neurotoxins 2,4-diaminobutyric acid [DAB], β-N-methylamino-L-alanine [BMAA] and N-(2-aminoethyl) glycine [AEG] in washings from plants collected from EGS fields; (c) quantifying DAB, BMAA and AEG in archived neural tissue from EGS and EMND horses; and (d) enumerating *Phormidium* spp. in washings of the biofilm on plants collected from fields grazed by horses which had idiopathic hepatopathy.

## Materials and methods

### Collection of plants

Most plants were collected from livestock-grazing fields (*n* = 88) in Scotland and North England. Most fields were grazed by horses (40 fields; termed horse control fields), cattle (13 fields) or sheep (14 fields) that had no apparent clinical neurological or hepatic disease, although detailed veterinary examinations were not performed. The remaining samples were collected from 21 “EGS” fields as soon as possible (always <96 h) after a horse grazing that field had developed acute EGS. EGS was confirmed by histopathology of autonomic and enteric ganglia [11]. Plants were collected by carefully cutting leaves/petioles with scissors approximately 1 cm above soil level, avoiding sampling of roots and adherent soil. To ensure that plant samples were representative of the entire field, samples comprised a mixture of ≥10 aliquots collected from points situated along an imaginary “W” transect, and comprised plants that were representative of the distribution of species present on the field. The varied mixtures of plants were typical of those growing on livestock grazing fields in Northern Britain. In horse fields, plants were collected only from grazed areas and not from non-grazed “roughs”. Samples were analysed within 4 h of collection, or were rapidly frozen within 30 min of collection by mixing with dry ice pellets and then stored in airtight polythene bags at −20 °C or −80 °C pending analysis.

To determine whether there was spatial variation in the density of *Phormidium* filaments within individual fields, samples were collected separately from 10 individual sites along the “W” transect in 2 fields, comprising one EGS field which had high overall *Phormidium* filament population density and one horse control field which had low overall density.

Two experiments were performed to determine whether there was temporal variation in the density of *Phormidium* filaments. To assess short-term variation, *Phormidium* filament population density was determined in samples collected from 9 fields (3 EGS, 6 horse control fields) at both 0700 and 1900 h. To assess longer term variation, *Phormidium* filament density was determined in samples collected from 8 fields at approximately weekly intervals from April to July 2006, including the period of highest incidence of EGS which is typically May in the UK [12]. One of these fields was a control horse field, while 7 were fields where at least one EGS case had occurred in the preceding 2 years; none of the fields had EGS cases during the sampling year. To determine if *Phormidium* filament density was influenced by weather, densities for the 8 individual fields were correlated with average daily air temperature, rainfall and sunshine hours, using data from a Meteorological Office weather station located within 48 km of all fields.

Unfortunately it was not possible to assess the population density of *Phormidium* filaments on fields grazed by EMND horses due to the rarity and sporadic occurrence of this disease [13]. Plants were also collected from 22 fields in France where the grazing horses had idiopathic subclinical hepatopathy, as evidenced by elevated serum activities of gamma glutamyltransferase and glutamate dehydrogenase. Despite extensive investigation, no aetiology had been identified. Samples were collected as described previously, but were kept on ice for 24 h before analysis.

### Enumeration of *Phormidium* filaments on plants

To suspend cyanobacteria that were adherent to plants, 20 mL sterile 0.9% saline was added to 10 g wet weight plants in a 50 mL plastic tube which was shaken vigorously by hand for 1 min. As a pilot study indicated that vigorous vortexing (Shaker VX-2500 Multi-Tube Vortexer, VWR International, Lutterworth, UK) of the plant suspension for 0, 1, 2 and 18 h had no significant effect on the yield of free filaments (data not presented), this method was not further employed. A 20 μL aliquot of the suspension was removed immediately after shaking, placed on a microscope slide, a cover-slip added and the total number of *Phormidium* filaments enumerated. The remaining suspension was frozen at −80 °C for future analysis.

### Detection of cyanobacterial 16S rDNA in plant washings, soil and equine ileal contents

To confirm identify of cyanobacteria within samples, 16S rDNA amplicons were prepared from plant washings from 6 EGS fields, soil from 1 EGS field and ileal contents from 2 EGS horses and sequenced using an Illumina MiSeq. Ileal contents were harvested within 2 h of death and stored at −80 °C pending analysis. Soil was collected from an EGS field, at a depth of 2–10 cm, and stored at −80 °C pending analysis. The MO-BIO Powersoil DNA Isolation Kit was used to extract total sample DNA following manufacturer’s instructions. Before bead beating, samples were heated at 65 °C for 10 min to increase cell lysis. 100 ng DNA was used in a two-round nested PCR protocol to amplify the V2-V3 region of the 16S gene. All PCR steps used the Q5 High-Fidelity 2X Master Mix (New England Biolabs). The first round of PCR consisted of 20 cycles using the primers 28 F (5’ GAGTTTGATCNTGGCTCAG 3’) and 805R (5’ GACTACCAGGGTATCTAATC 3’) in a total reaction volume of 50 μL. The reaction ran at 94 °C for 2 min, 20 cycles of 94 °C for 1 min, 55 °C for 45 s, 72 °C for 1.5 min followed by 72 °C for 20 min. After each PCR round, AMPure XP PCR Purification (Agencourt) was used to purify amplified DNA from other components of the reaction mixture. Purified PCR product (20 μL) was added into the second round of PCR along with the mastermix and barcoded primers 104 F (5’ GGCGVACGGGTGAGTAA 3’) and 519R (5’ GTNTTACNGCGGCKGCTG 3’) to a total volume of 50 μL. Primers include adapter sequences required for binding to the Illumina flow cell and barcodes for multiplexing. The reaction conditions were 98 °C for 30 s followed by 20 cycles of 98 °C for 10 s, 67 °C for 30 s, 72 °C for 10 s, and 72 °C for 2 min. 250 bp paired-end sequencing was performed using an Illumina Miseq.

### Bioinformatic analysis

Primers were removed using CUTADAPT [14] (allowing 1 bp error per 10 bp). MOTHUR [15] was used for quality control and for taxonomic assignment of reads, following a protocol developed for MiSeq by MOTHUR creators [16]. Sequences were removed from analysis if they were of length <350 bp, contained homopolymers of >9 bp or ambiguous base calls, did not align to the SILVA reference alignment [17], were identified as chimeric using UCHIME [18] or were classified as other than bacterial. Taxonomic classification was done using MOTHUR’s Bayesian classifier against the Greengenes database [19] trimmed to the V2-V3 16S region [20]. Sequences were assigned taxonomy if there was a >80% chance of correct assignment.

### Microscopic examination for cyanobacteria in equine gastrointestinal tract samples

To determine whether cyanobacteria were detectable microscopically in gastrointestinal contents of grazing horses, conventional light and fluorescence microscopy was performed on saline suspensions of freshly collected luminal contents from the stomach (*n* = 2), jejunum (*n* = 2), ileum (*n* = 4), caecum (*n* = 2) and colon (*n* = 2) of horses with acute EGS, and of faeces from 2 horses with chronic EGS and 2 healthy control horses.

### Analysis for DAB, BMAA and AEG in plant washings and equine neural tissue

DAB, BMAA and AEG were assayed using UPLC-MS/MS as previously described [21,22] in plant washing pellets from 3 EGS fields after 6 M HCl hydrolysis. The hydrolysate was centrifuge filtered, dried and derivatised with AQC for UPLC-MS/MS analysis, in comparison with synthetic standards [21]. Plant washing pellets were prepared by adding plants (5 g wet mass for sample 1, 6 g for samples 2 and 3) to 10 mL saline, shaking vigorously for 1 min, decanting the fluid, then ultracentrifugation at 13 000 rpm for 10 min before discarding the supernatant. These data were used to calculate the estimated daily DAB intake by a horse grazing for 24 h on each of these EGS fields.

DAB, BMAA and AEG were also assayed in archived formalin-fixed, wax-embedded, neural tissue from EGS and EMND-affected horses and from control horses. Sample 1 (EGS) comprised a pool of cranial cervical (superior) ganglia (CCG) from 6 different EGS-affected horses (mix breed and gender, median age 6 years, range 3–20). EGS was confirmed by histopathology of autonomic and enteric ganglia in all cases [11]. Sample 2 (control) comprised a pool of CCG from 6 different control horses (mix breed and gender; 14, 6–30 years) which were euthanased because of non-neurological disorders. Sample 3 (EMND) comprised pooled neural tissues from a 9 year-old Thoroughbred cross mare that had EMND confirmed at necropsy, including CCG, transverse segments of spinal cord at C1-2, C4-5, C7, C8, T1, T5, L6, S1 and rostral medulla oblongata. Sample 4 (EMND) was a sample of sacrococcygeus dorsalis muscle from a 6 year-old Cob gelding which had EMND. Sample 5 (control) was a wax-embedded formalin-fixed sample of medulla from a 20 year-old Thoroughbred cross mare that had no apparent neurologic disease. Neural samples were excised from formalin-fixed blocks, deparaffinised, washed with xylenes and hydrated using an alcohol series [23]. The subsequent material was hydrolysed with 6 M HCl for 16 h, the supernatant centrifuge filtered and dried in a speedvac. Once dry, the residue was resuspended with 20 mM HCl and derivatised with AQC for UPLC-MS/MS analysis of BMAA and its isomers.

### Statistical analysis

Data were not normally distributed. Inter- and within- group comparisons were made using the Mann Whitney and Wilcoxon Rank Sum tests, respectively. Correlations were done using Spearman’s Rank correlation. The spatial variability in *Phormidium* filament population density within a field was determined by calculating the coefficient of variance for 10 individual samples collected from that field.

## Results

### Identification of cyanobacteria in plant samples

**Figure 1:**
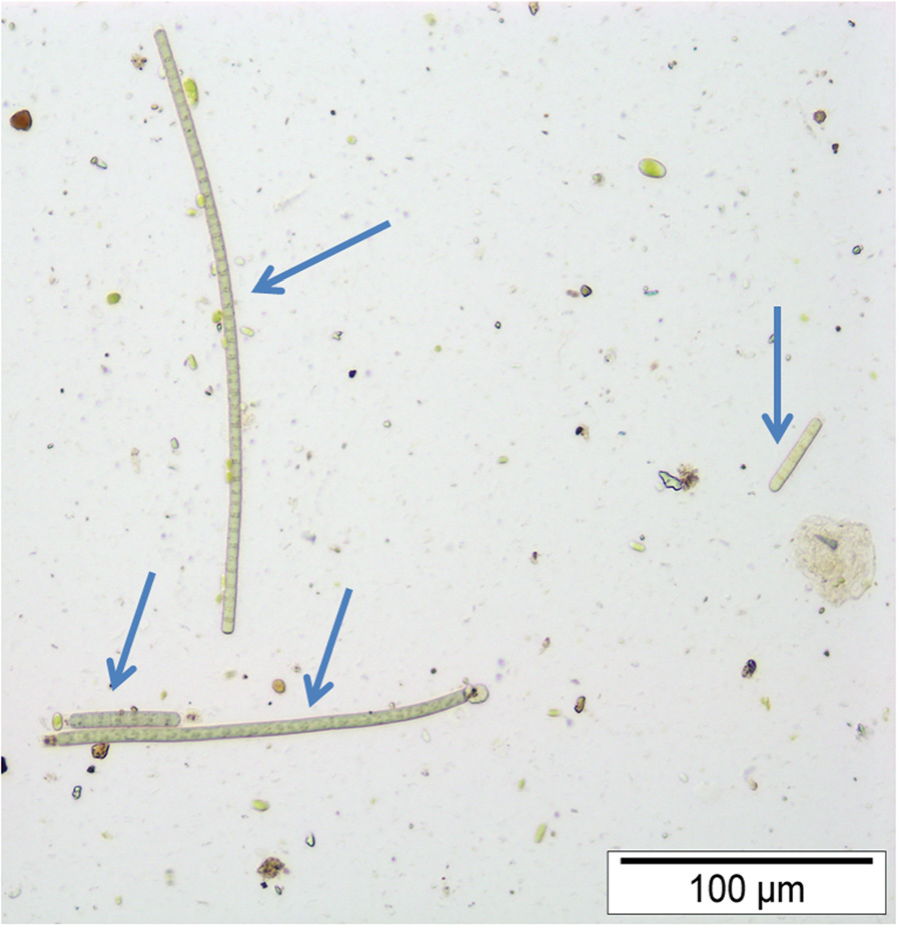
**Microscopic appearance of** ***Phormidium*** **filaments.** Four *Phormidium* filaments (arrows) in plant washings. Unstained specimen.

During sample collection it was noted that some plants, particularly at the base and branching points, had a slimy coating of mucilage consistent with that produced by cyanobacteria and algae [24]. Light and fluorescence microscopy revealed that these areas had a biofilm containing numerous cyanobacterial filaments. Microscopic examination of plant washings indicated that cyanobacteria were predominantly *Phormidium* spp. (Figure 1), with filaments in washings from many fresh samples exhibiting typical spontaneous oscillatory motion [25]. The numbers of cells per *Phormidium* filament on horse (median 16, range 3–67; *n* **=** 281), cattle (14, 6–56; *n* **=** 36) and sheep (18, range 4–76; *n* **=** 85) fields were not significantly different. Some plant samples also had low numbers of a second, very narrow filamentous cyanobacterium of the *Leptolyngbya* or *Leptothrix* genera, and low numbers of unicellular cyanobacteria of the *Aphanocapsa* genus. Many samples also had filamentous green algae, unicellular green algae, and low numbers of diatoms, motile algal flagellates and desmids (*Closterium* sp.): these were not enumerated.

### Density of *Phormidium* filaments in plant samples

**Figure 2:**
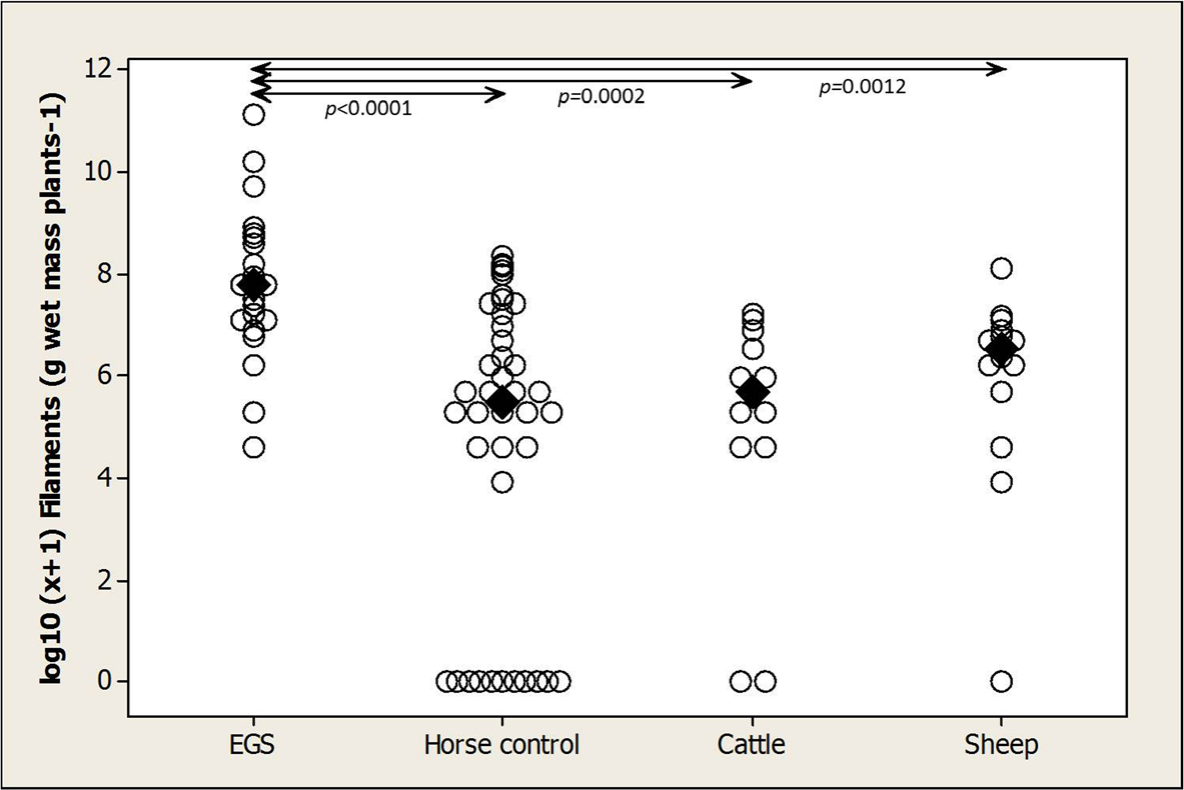
**Plants from EGS fields had significantly higher population densities of** ***Phormidium*** **filaments than plants from other fields.** Population density of *Phormidium* filaments (log_10_ [x + 1] g wet mass plants^−1^) on plants from EGS (*n* = 21), horse control (*n* = 40), cattle (*n* = 13) and sheep (*n* = 14) fields. Medians and inter-group significances are marked.

**Figure 3:**
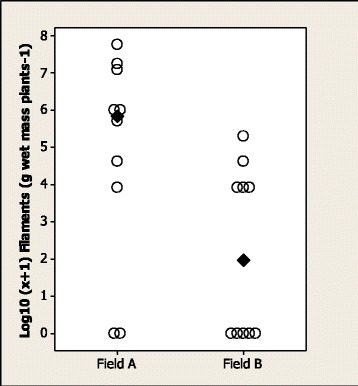
**Population densities of** ***Phormidium*** **filaments varied considerably, both within and between fields.** Spatial variation in density of *Phormidium* filaments (log_10_ [x + 1] g wet mass plants^−1^) across transects in an EGS field **(A)** and a horse control field **(B)**. Each datum point represents the density for each of 10 points across the field transect. Medians are marked.

**Figure 4:**
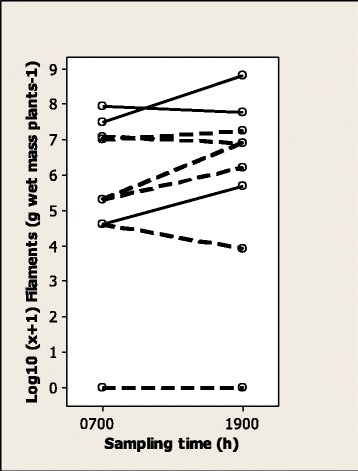
**Population densities of** ***Phormidium*** **filaments on plants collected at 0700 h and 1900 h did not differ significantly.** Comparison of the population density of *Phormidium* filaments (log_10_ [x + 1] g wet mass plants^−1^) in 9 fields (3 EGS fields with solid line, 6 horse control fields with broken line) in samples collected at 0700 h and 1900 h (data not significantly different). Medians are marked.

**Figure 5:**
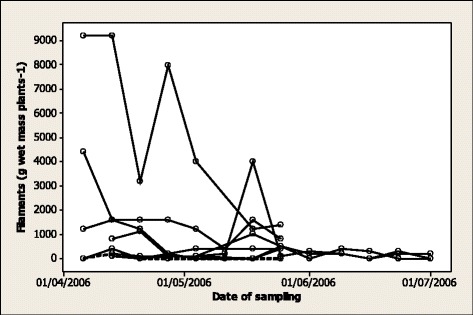
**Weekly variation in population density of** ***Phormidium*** **filaments on plants.** Weekly variation in population density of *Phormidium* filaments (g wet mass plants^−1^) on plants from one horse control (broken line) field and 7 fields (solid lines) on which there was at least one case of EGS within the preceding 2 years.

*Phormidium* filaments were present in samples from all EGS (*n* = 21) fields and most horse control (29/40), cattle (11/13) and sheep (13/14) fields. *Phormidium* filament population density was significantly higher in EGS fields (median 2400, range 100–68 900 filaments g wet mass plants^−1^) than in horse control (250, 0–4200; *p* < 0.0001), cattle (300, 0–1400; *p* = 0.0002) and sheep (700, 0–3300; *p* = 0.0012) fields (Figure 2). There were no significant differences in density among horse control, cattle and sheep fields. There was marked spatial variation in *Phormidium* filament population density across transects in an EGS field (coefficient of variance 126%) and a horse control field (143%) (Figure 3). There was marked variation, but no significant difference, in *Phormidium* filament population density in samples collected from 9 horse control fields at 0700 h and 1900 h, with density at 1900 h increased in 5 fields and reduced in 3 fields (Figure 4). There was no apparent consistent pattern to the weekly changes in population density in samples collected from 8 horse fields between April and July (Figure 5), and *Phormidium* filament population density in these samples was not significantly correlated with average air temperature, rainfall or sunshine hours. A low density (50, 50, 100 filaments g wet mass plants^−1^) of *Phormidium* filaments was present in only 3/22 samples from the French fields.

### Genomic identification of cyanobacteria in EGS plant washings, soil and equine ileal contents

Cyanobacterial 16S rDNA sequences were detected in all samples tested (see Additional file 1). Most sequences were attributable to unclassified *Phormidium*. The following sequences were identified; *Anabaena cylindrica* (1 plant washing), 1 unclassified *Nostoc* sp. (2 plant washings), 1 unclassified *Nostocaceae* fam. (2 plant washings), *Phormidium animale* (4 plant washings), 1 unclassified *Phormidium* sp. (6 plant washings, 2 ileal contents), 1 unclassified *Phormidium* sp. (2 plant washings), 1 unclassified *Phormidiaceae* fam. (2 plant washings), 1 unclassified *Oscillatoriales* ord. (1 plant washing), 1 unclassified *Oscillatoriophycideae* class (2 plant washings), *Leptolyngbya frigida* (1 plant washing), 1 unclassified *Leptolyngbya* sp. (1 plant washing), 1 unclassified *Pseudanabaenaceae* fam. (1 plant washing), 1 unclassified *Cyanobacteria* phylum (4 plant washings) and 1 unclassified *Cyanobacteria* phylum (3 plant washings). rDNA from 3 lines of *Melainabacteria* were identified; YS2/4C0d-2 (1 soil sample), mle1-12 (5 plant washings, 1 ileal contents, 1 soil sample) and ML635J-21 (5 plant washings, 1 soil sample). Additionally, rDNA from DAB-producing actinomycetes, including *Clavibacter* spp. and *Rathayibacter* spp., was detected in 6 plant washings and soil.

### Examination of cyanobacteria in equine gastrointestinal tract samples

No intact cyanobacteria were apparent. Gastric contents from 2 EGS horses appeared to contain occasional highly degraded and apparently non-viable *Phormidium* filaments.

### Analysis for DAB, BMAA and AEG

DAB was detected in all 3 plant washing pellets. Mean free and bound DAB concentrations (pg filament^−1^), respectively were: pellet 1, 43.5, 355.2; pellet 2, 121.6, 2131.4; pellet 3, 0.4, 8.2. BMAA and AEG were not detected in plant washings. BMAA, AEG and DAB were not detected in neural tissues at minimum detection limits 0.0065 picomoles per injection for L-BMAA and AEG and 0.0013 picomoles per injection for DAB.

### Estimation of the worst case scenario daily intake of *Phormidium* filaments and DAB for horses grazing EGS fields

Worst case scenario estimation of daily intake of *Phormidium* filaments for a horse grazing fulltime on the field with the highest *Phormidium* filament population density (68 900 filaments g wet weight plants^−1^) is 7.6 × 10^6^ filaments kg^−1^. This estimation assumes that an average 500 kg horse consumes 11.5 kg of herbage dry matter daily, with a typical mean dry matter of 21% [26], equating to 55 kg wet herbage daily. The estimated daily intakes of DAB (mg kg^−1^) for horses grazing the 3 EGS fields are: Field 1 (free 0.017; bound 0.14; total 0.16); Field 2 (free 0.007; bound 0.12; total 0.12); Field 3 (free 0.004; bound 0.08; total 0.09). Plant-washing pellets 1, 2 and 3 contained, respectively, 2700, 450 and 82 050 *Phormidium* filaments.

## Discussion

This is the first study to demonstrate that grazing livestock are exposed to pasture-derived terrestrial cyanobacteria, −microalgae (diatoms, *Closterium*) and their toxins. Indeed, the most commonly identified cyanobacteria, *Phormidium* spp., were identified within the biofilm of plants growing in most of the fields examined.

There was marked spatial variation in the population density of *Phormidium* on plants, both within and between fields, with counts ranging from 0–68 900 filaments g wet plants^−1^. Repeat sampling at 12 h and weekly intervals suggested there was also apparent marked temporal variation in *Phormidium* population density, although some of this variability could have reflected spatial variation. *Phormidium* density did not appear to follow a consistent diurnal or weekly pattern and was not apparently correlated with average daily air temperature, rainfall or sunshine hours. However these latter findings should be interpreted with caution since these relationships were assessed under limited circumstances and further detailed investigation is required to clarify the influence of weather on *Phormidium* density. Variation in *Phormidium* population density presumably reflects the suitability of the local biofilm microenvironment for cyanobacterial growth and survival. Factors reported to influence cyanobacterial growth and survival include light duration and irradiance, temperature, availability of water, phosphate-, iron- and nitrate concentrations, and numbers of invertebrate prey species [27,28]. We hypothesise that high exposure levels may be encountered when environmental conditions are optimal, as occurs on turf grasses [2-6], a situation akin to the occurrence of cyanobacterial blooms in aquatic environments during eutrophication [29,30]. Indeed the highest *Phormidium* population density recorded in the study (68 900 filaments g wet plants^−1^) was a clear statistical outlier suggesting that it could be considered to represent such a “bloom” occurrence. Variation in apparent *Phormidium* density also likely reflects the organism’s motility, with filaments moving up and down plant leaves/petioles at up to 11 μm s^−1^ [31], presumably to seek favourable light conditions. As *Phormidium* spp. are more likely to be present at the tips of short plants than long plants [5], *Phormidium* population density within plant washings may also reflect plant length and the proportion of lower and upper leaves/petioles sampled. This study may have underestimated *Phormidium* density because an indeterminate number of cyanobacteria may have remained adherent within the plant biofilm despite vigorous shaking. While the *Phormidium* spp. were readily enumerated in plant washings, many of the aforementioned factors which influence *Phormidium* population density will compromise accurate estimation of the number of *Phormidium* filaments ingested by grazing animals. For example, since there was considerable spatial and temporal variation in cyanobacterial density, the number of cyanobacteria ingested may be markedly higher in an animal grazing a cyanobacterium-rich area of grassland. In a worst-case scenario estimation, a 500 kg horse grazing fulltime on the EGS field with the highest *Phormidium* population density (68 900 filaments g wet mass plants^−1^) and a median number of 16 cells filament^−1^, would ingest 7.6 × 10^6^*Phormidium* filaments kg^−1^ day^−1^or 121 × 10^6^*Phormidium* cells kg^−1^ day^−1^.

Ingestion of terrestrial cyanobacteria provides one explanation for the presence of cyanobacterial 16S rDNA in the 2 equine ileal samples in this study. It also provides a more plausible explanation for the previous report of cyanobacterial 16S rDNA in faeces from forage-fed horses than them being homologous sequences from dietary plant chloroplasts [32]. Alternative explanations include ingestion of aquatic cyanobacteria from contaminated water sources and ingestion of foods containing cyanobacterial supplements. Cyanobacterial 16S rDNA sequences detected in ileal contents was attributable to unclassified *Phormidium* spp. (2 horses) and a bacterium of the Order mle1-12 (1 horse). The latter has recently been proposed as a member of a candidate class [33] or sister phylum [34] of non-photosynthetic, filamentous bacteria termed *Melainabacteria,* distinct from extant cyanobacteria, but with ancestral affiliations. The fate of ingested cyanobacteria is currently receiving increasing attention. The ability of ingested cyanobacteria to colonise the gastrointestinal tract has so far received little attention. Indeed cyanobacterial species in aquatic environments do not typically thrive below about pH 6.7 and cell degradation can occur under acid conditions (Codd, personal observations). Intact cyanobacteria were not identified microscopically in the equine gastrointestinal contents, although gastric contents of 2 EGS horses appeared to contain occasional highly degraded and apparently non-viable *Phormidium* filaments. Similarly cyanobacteria in the rumen and abomasum of cattle dying from acute cyanobacterial poisoning are markedly degraded (Codd, personal observations). While spores (akinetes) produced by some cyanobacteria could potentially survive in the gut, akinete production is not widespread among cyanobacteria and does not occur in *Phormidium* which predominated in this study. Further, it is not known whether akinete germination and outgrowth could occur in the aphotic, anaerobic gut. However, it is known that some species of *Phormidium* (*P. uncinatum*) can grow chemoheterotrophically (aphotic growth on glucose, fructose and acetate at a reduced rate) [35]. Furthermore, recent work indicates that the unicellular aquatic cyanobacterium *Microcystis* PCC7806 can survive and potentially produce cyanotoxins for up to 17 days in an in vitro environment mimicking the human gastrointestinal tract [36]. The possibility of gut colonisation by cyanobacteria is further supported by accumulating evidence that *Melainabacteria,* including the mle1-12 present in 1 horse ileum, have differentiated from extant cyanobacteria by niche adaptation, including for symbiosis in the mammalian gut [34,37-39]. Indeed, *Melainabacteria* is considered to have an obligate fermenter role in the gut, and its enrichment in herbivores may reflect a prominent role in plant fibre digestion [34]. Further work, utilising larger numbers of horses, is required to characterise the populations of cyanobacteria within the equine gastrointestinal tract, and to determine whether they are capable of colonisation and in vivo cyanotoxin production. If so, it would add support to the hypothesis that cyanobacteria, while being typically a minor component of the intestinal microflora, may proliferate and produce neurotoxins in vivo, resulting in neurodegenerative diseases [40].

Further study is warranted to identify and quantify the toxins produced by *Phormidium* from livestock fields and to determine whether under appropriate environmental conditions, grazing livestock can ingest sufficient quantities of cyanotoxins from terrestrial cyanobacteria to cause disease. While the range of toxins produced by *Phormidium* is currently unknown, production of BMAA, LPS, microcystins and anatoxin-a is reported [8,9,41-43]. Extrapolation of data from the related cyanobacterial genus *Oscillatoria* suggests that *Phormidium* may also produce, apslysiatoxins, cylindrospermopsin and homoanatoxin-a. These toxins cause hepatotoxicity, neurotoxicity and dermatitis in animals exposed to aquatic cyanobacterial blooms [7-10]. BMAA has been linked with human motor neuron disease, Alzheimer’s disease and Parkinson’s disease [44-48] and proposed as a potential cause of equine motor neuron disease [40]. The design of this study precluded definitive examination of potential associations between ingested cyanotoxins and disease in grazing animals. Since livestock grazing the horse control-, cattle- and sheep fields had no overt evidence of hepatic or neurological disease (although veterinary examinations were not performed), cyanotoxin exposure in these fields must have been insufficient to cause clinical disease. Additional experiments were performed to test the hypothesis that, under appropriate circumstances, ingestion of cyanobacterial hepato- and neurotoxins contributes to the pathogenesis of some currently unexplained diseases of grazing horses, including EGS, EMND of grazing horses [13] and hepatopathy.

*Phormidium* population density was significantly higher on EGS fields than on control fields, indicating that horses grazing EGS fields likely ingest higher numbers of *Phormidium* spp. than horses grazing control fields. This brings into question conclusions of a previous study [26] that cyanotoxins can be excluded as a causative factor for EGS. While the present study assessed exposure of horses to terrestrial cyanobacteria, the previous study [26] investigated only exposure to aquatic cyanobacteria, finding no detectable microcystins and no microscopic evidence of cyanobacteria in water samples from 16 premises on which EGS had occurred. While the cause of EGS is unknown, increasing evidence suggests it is a toxico-infectious form of botulism, whereby a dietary trigger induces intestinal overgrowth of *Clostridium botulinum* C and/or D, with resultant in vivo production of botulinum neurotoxins (reviewed in [49]). Potential involvement of cyanotoxins in EGS could therefore reflect their proposed action as triggers for botulism [50-52] or reflect direct neurotoxic effects of cyanotoxins including microcystins, anatoxin-a, DAB and BMAA [1,8,53]. Alternatively the increased *Phormidium* density on EGS fields may be unrelated to EGS pathogenesis, perhaps simply reflecting the elevated total nitrogen and ammonium nitrogen content of soils on EGS fields [26,54], since nitrate promotes growth of some cyanobacteria [55].

Since DAB, BMAA and AEG were not detected in neural tissues from 6 EGS, 2 EMND horses and 7 control horses, a causal role for these neurotoxins in EGS and EMND could not be demonstrated. DAB was however detected in all washing pellets of plants from 3 EGS fields. While DAB is produced by cyanobacteria, additional sources include plant associated actinomycetes and some leguminous plants [56,57]. Indeed the apparent lack of correlation between DAB concentrations and the concentration of *Phormidium* filaments in the 3 plant washing pellets suggests that DAB was derived from a source other than, or in addition to, *Phormidium*. One further potential source of DAB in grass washings is actinomycetes, including the plant pathogenic *Clavivibacter* spp. and *Rathayibacter* spp., since 16S rDNA sequences from these bacteria were detected in 6 plant washings and soil. While the effect of ingested DAB on horses is unknown, the estimated worse-case scenario daily intake of DAB (free 0.017; bound 0.14; total 0.16 mg kg^−1^) is lower than doses used experimentally to induce neurolathyrism in laboratory animals (from 0.7 to approximately 500 mg kg^−1^ [58-60]).

*Phormidium* was present only in low numbers in plants collected from fields in France where horses had unexplained hepatopathy, indicating that a role in disease aetiology was unlikely.

This is the first study to demonstrate that grazing livestock are exposed to terrestrial cyanobacteria. While it did not yield evidence linking terrestrial cyanotoxins with neurologic or hepatic disease in grazing horses, further study is required to identify and quantify cyanotoxin exposure in grazing livestock, and to determine whether, under appropriate conditions, terrestrial cyanotoxins contribute to currently unexplained diseases. Additional study is also warranted to further identify cyanobacteria within the gastrointestinal tract of herbivores and determine whether some are capable of intestinal colonisation and in vivo toxin production.

## Additional file

Additional file 1:
**Identity of cyanobacterial 16S rDNA sequences detected in EGS plant washings (** ***n*** **= 6), equine ileal contents (** ***n*** **= 2) and soil (** ***n*** **= 1).** Numbers in parentheses refer to numbers of plant washings (P), ileal contents (I) and soil (S) which contained each sequence.

## Abbreviations

AEG: N-(2-aminoethyl) glycine
BMAA: β-N-methylamino-L-alanine
CCG: Cervical [superior] ganglia
DAB: 2,4-diaminobutyric acid
EGS: Equine grass sickness
EMND: Equine motor neuron disease

## References

[1] Codd GA, Lindsay J, Young FM, Morrison LF, Metcalf JS, Huisman J, Matthijs HCP, Visser PM (2005). Cyanobacterial Toxins. Harmful Cyanobacteria.

[2] Baldwin NA, Whitton BA (1992). Cyanobacteria and eukaryotic algae in sports turf and amenity grasslands: a review. J App Phycol.

[3] Hodges CF, Campbell DA (1997). Nutrient salts and the toxicity of black-layer induced by cyanobacteria and *Desulfovibrio desulfuricans* to *Agrostis palustris*. Plant Soil.

[4] Elliott ML (1998). Use of fungicides to control blue-green algae on Bermuda grass putting-green surfaces. Crop Protection.

[5] Gelernter W, Stowell LJ (2000). Cyanobacteria (A.K.A. blue-green algae): wanted for causing serious damage to turf. PACE Insights.

[6] Tredway LP, Stowell LJ, Gelertner WD (2006). Yellow spot and the potential role of cyanobacteria as turf grass pathogens. Golf course management.

[7] Skulberg OM, Carmichael WW, Andersen RA, Matsunaga S, Moore RE, Skulberg R (1992). Investigations of a neurotoxic Oscillatorialean strain (cyanophyceae) and its toxin. Isolation and characterization of homoanatoxin-a. Environ Toxicol Chem.

[8] Cox PA, Banack SA, Murch SJ, Rasmussen U, Tien G, Bidigare RR, Metcalf J, Morrison LF, Codd JA, Bergman B (2005). Diverse taxa of cyanobacteria produce-N-methylamino-L-alanine, a neurotoxic amino acid. Proc Natl Acad Sci U S A.

[9] Gugger M, Lenoir S, Berger C, Ledreux A, Druart JC, Humbert JF, Guette C, Bernard C (2005). First report in a river in France of the benthic cyanobacterium *Phormidium favosum* producing anatoxin-a associated with dog neurotoxicosis. Toxicon.

[10] van Apeldoorn ME, van Egmond HP, Speijers GJA, Bakker GJI (2007). Toxins of cyanobacteria. Mol Nutr Food Res.

[11] Doxey DL, Pogson DM, Milne EM, Gilmour JS, Chisholm HK (1992). Clinical equine dysautonomia and autonomic neuron damage. Res Vet Sci.

[12] Doxey DL, Gilmour JS, Milne EM (1991). A comparative study of normal equine populations and those with grass sickness (dysautonomia) in eastern Scotland. Equine Vet J.

[13] McGorum BC, Mayhew IG, Amory H, Deprez P, Gillies L, Green K, Mair TS, Nollet N, Wijnberg I, Hahn C (2006). Horses on pasture may be affected by equine motor neuron disease. Equine Vet J.

[14] Martin M (2011). Cutadapt removes adapter sequences from high-throughput sequencing reads. EMBnet J.

[15] Schloss PD, Westcott SL, Ryabin T, Hall JR, Hartmann M, Hollister EB, Lesniewski RA, Oakley BB, Parks DH, Robinson CJ, Sahl JW, Stres B, Thallinger GG, Van Horn DJ, Weber CF (2009). Introducing mothur: open-source, platform-independent, community-supported software for describing and comparing microbial communities. Appl Environ Microbiol.

[16] Kozich JJ, Westcott SL, Baxter NT, Highlander SK, Schloss PD (2013). Development of a dual-index sequencing strategy and curation pipeline for analyzing amplicon sequence data on the MiSeq illumina sequencing platform. Appl Environ Microbiol.

[17] Pruesse E, Quast C, Knittel K, Fuchs BM, Ludwig W, Peplies J, Glöckner FO (2007). SILVA: a comprehensive online resource for quality checked and aligned ribosomal RNA sequence data compatible with ARB. Nucleic Acids Res.

[18] Edgar RC, Haas BJ, Clemente JC, Quince C, Knight R (2011). UCHIME improves sensitivity and speed of chimera detection. Bioinformatics.

[19] DeSantis TZ, Hugenholtz P, Larsen N, Rojas M, Brodie EL, Keller K, Huber T, Dalevi D, Hu P, Andersen GL (2006). Greengenes, a chimera-checked 16S rRNA gene database and workbench compatible with ARB. Appl Environ Microbiol.

[20] Werner JJ, Koren O, Hugenholtz P, DeSantis TZ, Walters WA, Caporaso JG, Angenent LT, Knight R, Ley RE (2012). Impact of training sets on classification of high-throughput bacterial 16 s rRNA gene surveys. ISME J.

[21] Banack SA, Metcalf JS, Jiang L, Craighead D, Ilag LL, Cox PA (2012). Cyanobacteria produce N-(2-Aminoethyl)Glycine, a backbone for peptide nucleic acids which may have been the first genetic molecules for life on Earth. PLoS One.

[22] Masseret E, Banack SA, Boumediene F, Abadie E, Brient L, Pernet F, Juntas-Morales R, Pageot N, Metcalf J, Cox P, Camu W (2013). The French network on BMAA/ALS. Detection of BMAA in the marine environment of an ALS cluster in Southern France. PLoS One.

[23] Scicchitano MM, Dalmas DA, Boyce RW, Thomas HC, Frazier KS (2009). Protein extraction of formalin-fixed, paraffin-embedded tissue enables robust proteomic profiles by mass spectrometry. J Histochem Cytochem.

[24] Sutherland IW (1996). A natural terrestrial biofilm. J Indust Microbiol.

[25] Richardson LL, Castenholz RW (1989). Chemokinetic motility responses of the cyanobacterium *Oscillatoria terebriformis*. Appl Environ Microbiol.

[26] Edwards SE, Martz KE, Rogge A, Heinrich M (2010). Edaphic and phytochemical factors as predictors of equine grass sickness cases in the UK. Front Pharmacol.

[27] Mur LR (1983). Some aspects of the ecophysiology of cyanobacteria. Ann Microbiol.

[28] Agrawal SC, Singh V (2002). Viability of dried filaments, survivability and reproduction under water stress, and survivability following heat and UV exposure in *Lyngbya martensiana, Oscillatoria agardhii, Nostoc calcicola, Hormidium fluitans, Spirogyra sp.* and *Vaucheria geminate*. Folia Microbiol.

[29] Fogg GE, Stewart WDP, Fay P, Walsby AE (1972). The Blue-Green Algae.

[30] Sutcliffe DW, Jones JG (1992). *Eutrophication*: *Research and Application to Water Supply*.

[31] Staley JT (1986). Bergey’s Manual of Systematic Bacteriology.

[32] Shepherd ML, Swecker WS, Jensen RV, Ponder MA (2012). Characterization of the fecal bacterial communities of forage-fed horses by pyrosequencing of 16S rRNA V4 gene amplicons. FEMS Microbiol Lett.

[33] Soo RM, Skennerton CT, Sekiguchi Y, Imelfort M, Paech SJ, Dennis PG, Steen JA, Parks DH, Tyson GW, Hugenholtz P (2014). An expanded genomic representation of the phylum Cyanobacteria. Genome Biol Evol.

[34] Di Rienzi SC, Sharon I, Wrighton KC, Koren O, Hug LA, Thomas BC, Goodrich JK, Bell JT, Spector TD, Banfield JF, Ley RE (2013). The human gut and groundwater harbor nonphotosynthetic bacteria belonging to a new candidate phylum sibling to Cyanobacteria. Elife.

[35] Bagchi SN, Chauhan VS, Palod A (1990). Heterotrophy and nitrate metabolism in a cyanobacterium *Phormidium uncinatum*. Curr Microbiol.

[36] Stefanelli M, Vichi S, Stipa G, Funari E, Testai E, Scardala S, Manganelli M (2014). Survival, growth and toxicity of *Microcystis aeruginosa* PCC 7806 in experimental conditions mimicking some features of the human gastro-intestinal environment. Chem Biol Interact.

[37] Backhed F, Ley RE, Sonnenburg JL, Peterson DA, Gordon JI (2005). Host-bacterial mutualism in the human intestine. Science.

[38] Eckburg PB, Bik EM, Bernstein CN, Purdom E, Dethlefsen L, Sargent M, Gill SR, Nelson KE, Relman DA (2005). Diversity of the human intestinal microflora. Science.

[39] Ley R, Backhed F, Turnbaugh P, Lozupone C, Knight R, Gordon J (2005). Obesity alters gut microbial ecology. Proc Natl Acad Sci U S A.

[40] Brenner SR (2013). Blue-green algae or cyanobacteria in the intestinal micro-flora may produce neurotoxins such as Beta-N-Methylamino-L-Alanine (BMAA) which may be related to development of amyotrophic lateral sclerosis, Alzheimer’s disease and Parkinson-Dementia-Complex in humans and Equine Motor Neuron Disease in horses. Med Hypotheses.

[41] Mikheyskaya LV, Ovodova RG, Ovodov YS (1977). Isolation and characterization of lipopolysaccharides from cell walls of blue-green algae of the genus *Phormidium*. J Bacteriol.

[42] Codd GA, Bell SG, Kaya K, Ward CJ, Beattie KA, Metcalf JS (1999). Cyanobacterial toxins, exposure routes and human health. Eur J Phycol.

[43] Aboal M, Puig MA, Asencio AD (2005). Production of microcystins in calcareous Mediterranean streams: the Alharabe River, Segura River basin in south-east Spain. J Appl Phycol.

[44] Spencer PS, Nunn PB, Hugon J, Ludolph AC, Ross SM, Roy DN, Robertson RC (1987). Guam amyotrophic lateral sclerosis-Parkinsonism-dementia linked to a plant excitant neurotoxin. Science.

[45] Cox PA, Banack SA, Murch SJ (2003). Biomagnification of cyanobacterial neurotoxins and neurodegenerative disease among the Chamorro people of Guam. Proc Natl Acad Sci U S A.

[46] Bradley WG, Mash DC (2009). Beyond Guam: The cyanobacteria/BMAA hypothesis of the cause of ALS and other neurodegenerative diseases. Amyotroph Lat Scler.

[47] Pablo J, Banack SA, Cox PA, Johnson TE, Papapetropoulos S, Bradley WG, Buck A, Mash DC (2009). Cyanobacterial neurotoxin BMAA in ALS and Alzheimer’s disease. Acta Neurol Scand.

[48] Chiu AS, Gehringer MM, Welch JH, Neilan BA (2011). Does α-amino-β-methylaminopropionic acid (BMAA) play a role in neurodegeneration?. Int J Environ Res Public Health.

[49] Newton JR, Wylie CE, Proudman CJ, McGorum BC, Poxton IR, Overviews S (2010). Equine grass sickness: are we any nearer to answers on its cause and prevention after a century of research?. Equine Vet J.

[50] Eriksson JE, Lindholm T. Sinilevakukintojen aiheuttamat lintu-ja kalakuolemat. Vesistojen levahaitat. Maakunnallinen vesipaiva. Lahtis Motespublikation. 1998; May 18.

[51] Mahakhant A, Klungsugya P, Arunpairojana A, Sano T, Watanabe M, Kaya K, Atthasampunna P (1999) Toxicity of cyanobacterial blooms in Thailand. Thailand Institute of Scientific and Technological Research; Research Project No. 39–02

[52] Murphy T, Lawson A, Nalewajko C, Murkin H, Ross L, Oguma K, McIntyre T (2000). Algal toxins - initiators of avian botulism?. Inc Environ Toxicol.

[53] Menga G, Suna Y, Fua W, Guob Z, Lihong X (2011). Microcystin-LR induces cytoskeleton system reorganization through hyperphosphorylation of tau and HSP27 via PP2A inhibition and subsequent activation of the p38 MAPK signaling pathway in neuroendocrine (PC12) cells. Toxicol.

[54] McCarthy HE, French NP, Edwards GB, Miller K, Proudman CJ (2004). Why are certain premises at increased risk of equine grass sickness? A matched case–control study. Equine Vet J.

[55] Kumar Saha S, Uma L, Subramanian G (2003). Nitrogen stress induced changes in the marine cyanobacterium *Oscillatoria willei* BDU 130511. FEMS Microbiol Ecol.

[56] Sasaki J, Chijimatsu M, Suzuki K (1998). Taxonomic significance of 2,4-diaminobutyric acid isomers in the cell wall peptidoglycan of actinomycetes and reclassification of *Clavibacter toxicus* as *Rathayibacter toxicus* comb. nov. Int J Syst Bacteriol.

[57] Kruger T, Monch B, Oppenhauser S, Luckas B (2010). LC–MS/MS determination of the isomeric neurotoxins BMAA (b-N-methylamino-L-alanine) and DAB (2,4-diaminobutyric acid) in cyanobacteria and seeds of *Cycas revoluta* and *Lathyrus latifolius*. Toxicon.

[58] Ressler C, Redstone PA, Erenberg RH (1961). Isolation and identification of a neuroactive factor from *Lathyrus latifolius*. Science.

[59] Mushahwar IS, Koeppe RE (1963). Concerning the metabolism of D- and L-α, γ-diaminobutyric acid-2–C14 in rats. J Biol Chem.

[60] Vivanco F, Ramos F, Jimenez-Diaz C (1966). Determination of γ-aminobutyric acid and other free amino acids in whole brains of rats poisoned with β, β’iminodipropionitrile and α, γ- diaminobutyric acid with, or without, administration of thyroxine. J Neurochem.

