# Supplementary material for: Grazing livestock are exposed to terrestrial cyanobacteria
Source: Vet Res. 2015 Feb 25;46:16. doi: 10.1186/s13567-015-0143-x (PMC4342207; doi:10.1186/s13567-015-0143-x)
Supplement: Additional file 1: — Identity of cyanobacterial 16S rDNA sequences detected in EGS plant washings ( n = 6), equine ileal contents ( n = 2) and soil ( n = 1). Numbers in parentheses refer to numbers of plant washings (P), ileal contents (I) and soil (S) which contained each sequence. [file 13567_2015_143_MOESM1_ESM.pdf]

**CLASS**	**ORDER**	**FAMILY**	**GENUS**	**SPECIES**
Unclassified	unclassified	unclassified	unclassified	unclassified (3P)
4Cod-2	MLE1-12	unclassified	unclassified	unclassified (5P, 1I, 1S)
	YS2	unclassified	unclassified	unclassified (1S)
ML635J-21	unclassified	unclassified	unclassified	unclassified (5P, 1S)
Nostocophycideae	Nostocales	Nostocaceae	Anabaena	cylindrica (1P)
			Nostoc	unclassified (2P)
			unclassified	unclassified (2P)
Oscillatoriophycideae	Oscillatoriales	Phormidiaceae	Phormidium	unclassified (6P, 2I)
				animale (4P)
				unclassified (2P)
			unclassified	unclassified (2P)
		unclassified	unclassified	unclassified (1P)
	unclassified	unclassified	unclassified	unclassified (2P)
Synechococcophycideae	Pseudanabaenales	Pseudanabaenaceae	Leptolyngbya	unclassified (1P)
				frigida (1P)
			unclassified	unclassified (1P)
unclassified	unclassified	unclassified	unclassified	unclassified (4P)
